# Traditional Uses of Medicinal Plants by Ethnic People in the Kavrepalanchok District, Central Nepal

**DOI:** 10.3390/plants9060759

**Published:** 2020-06-17

**Authors:** Gabriele Ambu, Ram Prasad Chaudhary, Mauro Mariotti, Laura Cornara

**Affiliations:** 1 Department of Earth, Environment and Life Sciences, University of Genova, 16132 Genova, Italy; frategabriele@libero.it (G.A.); m.mariotti@unige.it (M.M.); 2 Research Centre for Applied Science and Technology (RECAST), Tribhuvan University, 44613 Kathmandu, Nepal; ram.chaudhary53@gmail.com

**Keywords:** traditional ecological knowledge, ethnopharmacology, medicinal plants, conservation

## Abstract

In rural areas of Nepal, where it is difficult to get access to Government health care facilities, people depend on medicinal plants and local healers for health problems. This study concerns an ethnobotanical survey of the Kavrepalanchok District, reporting some unusual uses of medicinal plants and original recipes. A total of 32 informants were interviewed, 24 of them being key informants. Ethnobotanical uses concerned 116 taxa, of which 101 were medicinal plants, with the most representative species belonging to Asteraceae, Fabaceae, Lamiaceae, and Zingiberaceae. Ethnobotanical indexes were used to evaluate the ethnopharmacological importance of each plant species and the degree of agreement among the informants’ knowledge. Informant consensus factor (Fic) showed that the fever category had the greatest agreement. Highest fidelity level (FL) values were found for *Calotropis gigantea* used for dermatological diseases, *Drymaria cordata* for fever, *Mangifera indica* and *Wrightia arborea* for gastrointestinal disorders. Data document the richness of the local flora and the traditional knowledge on medicinal plant species used by ethnic communities in rural areas. The active involvement of local populations in the conservation and management of medicinal plant species will encourage future projects for the sustainable development of the biological and cultural diversity of these rural areas of Nepal.

## 1. Introduction

Traditional systems of medicine are important health sources spread all over the world, especially in developing countries [1]. Most interesting ethnobotanical data can be generally collected in ethnic communities living in rural areas of remote regions, where Traditional Ethnobotanical Knowledge (TEK) remains often underdocumented without a proper documentation [2]. In such contexts it is pivotal to preserve the interaction between indigenous peoples and their environment to avoid that this knowledge fades out in a few generations. Accordingly, it appears increasingly important the role of TEK for promoting sustainable ecosystems and to develop strategies for protecting and enhancing the natural resources.

In Nepal, out of a total of approximately 28 million inhabitants, 80% lives in rural areas [3,4], where it is difficult to access government health care facilities. It is estimated that there are only 2 physicians per 10,000 people, while in other parts of the world the number is higher, e.g. in Europe there are on average 33 physicians per 10,000 people (minimum Romania 19, maximum Greece 54) [5]. After the earthquake (8 M_w_) in April 2015, access to medical care has became even more problematic and rural areas have been exposed to many epidemic diseases, especially among children and elderly people. Therefore, people in these areas depend highly on traditional use of medicinal plants for their primary health care. This traditional knowledge, passed down orally mainly within families or small groups of healers, includes folk, shamanistic and Ayurvedic medicine [6].

Due to its significant variations in altitude, topography and climate, Nepal has an important floral biodiversity with 6500 species of flowering plants and ferns [7] of which 2000 are commonly used in traditional healing practices [8]. Also there is a high diversity in ethnic groups (125), each of them with its own culture, language, religious rites, and traditional practices in the use of medicinal plants [9,10].

The present study is an in-depth investigation of medicinal plants used by ethnic people residing in several villages of Kavrepalanchok District, which are located outside tourist circuits and characterized by a high rate of poverty. Our results integrate previous ethnobotanical studies conducted in this zone of Central Nepal [11,12,13,14,15,16,17,18,19,20,21,22,23,24,25,26,27], with a special focus on medicinal plants selected and used by local healers and shamans who are considered the depository of TEK. The data highlight most quoted species in the treatment of specific pathologies, as shown by ethnobotanical indexes. In addition, we have also recorded some unusual uses of medicinal plants and original recipes, as well as the use of species that have never been reported in previous ethnobotanical studies from Central Nepal. The valorisation of folk medicine can promote a sustainable development of the natural resources in these rural areas.

## 2. Results

A total of 32 informants (26 men and 6 women) aged between 23 and 81 years were interviewed (Table 1).

About 38% were illiterate (12 informants), 34% had a primary level of literacy (11 informants), about 25% a secondary level (8 informants) and only one informant (3%) had a degree. Among them, 24 were selected as key informants as follows: 8 shamans (*jhankris*), 2 local healers, 14 farmers and plant traders. The interviewed shamans belonged to the Tamang ethnic group (one was a woman) and the local healers belonged to the Brahmin ethnic group and were experts in Ayurvedic medicine. These informants lived in remote areas and preserved old tradition. In our survey, ethnobotanical knowledge was concentrated among informants with primary education (41%), followed by those with secondary education (33%), and finally by those who had no educational level (22%) while the only one graduate informant corresponded to 4% of the total. The 6 women provided 15% of the information collected.

### 2.1. Plants Diversity

The informants reported 318 ethnobotanical uses of 116 plants belonging to 57 families (Table 2).

The taxonomic diversity percentage was calculated: the most representative species belonged to Asteraceae (10.34%), Fabaceae (6.03%), Lamiaceae (5.17%) and Zingiberaceae (4.31%) (Table 3).

Among them the most representative were herbs (36.20%), followed by trees (26.72%), shrubs (25%), climbers (8.62%) and parasitic plants species (1.72%); further, two ferns were also recorded (1.72%). These data are indicative of the richness of the local flora and testify the botanical knowledge of the informants, in accordance with previous studies conducted in Central Nepal [28,29].

### 2.2. Ethnomedicinal Uses of Plants

**Figure 1:**
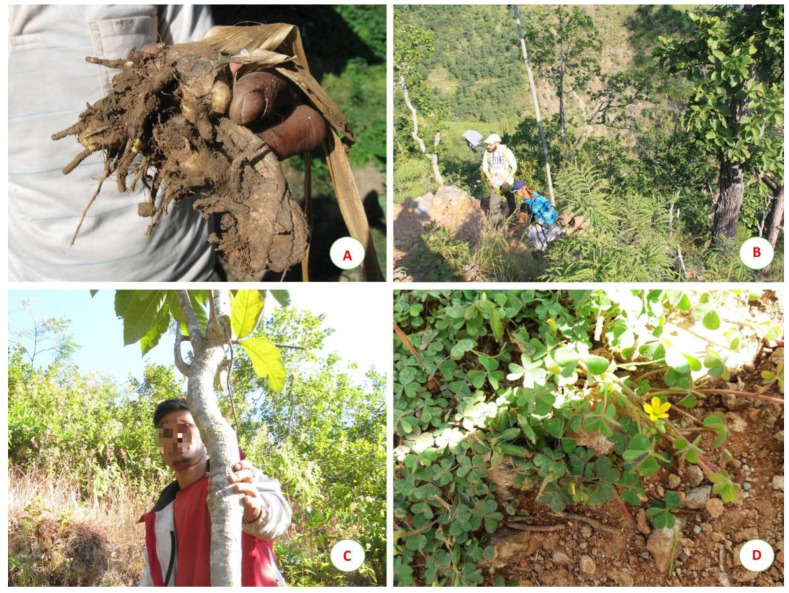
(**A**) Rhizome of *Cautleya spicata*; (**B**) Medicinal plants search during a jungle walk around Mukpatar village; (**C**) *Ficus semicordata* used for gastric problems; (**D**) *Oxalis corniculata,* remedy for the treatment of various diseases.

About 87% of the 116 species (101) was reported for medicinal purposes, with 271 citations of uses. Among these species, 20 were reported also in other categories, particularly as food and food/medicine (11) and religious/ritual (6). Minor uses concerned domestic, handcraft, and agropastoral categories. The plants used were mostly wild plants easy to find, in particular, herbs, trees and shrubs growing near villages. Sometimes even some cultivated plants were used for medicinal purposes (Figure 1A).

More rarely, hard-to-find plants were also selected, such as *Viscum articulatum*, *Piper retrofractum* and *Picrasma quassioides* that grow in inaccessible areas of the forest (Figure 1B). Medicinal plants were used by informants to treat 13 categories of human ailments and 1 related to cattle diseases (Table 4); 48 species were used to treat only one disease (e.g., *Ficus semicordata*, Figure 1C) and 53 species to treat more than one ailment (e.g., *Oxalis corniculata*, Figure 1D).

The most frequent disease categories treated with medicinal plants and showing the highest citations, were those concerning fever, digestive system, skeletal and muscular system, and skin diseases (56, 45, 30 and 25 citations, respectively). Traditional healers identified the diseases according to traditional patient examination, including inspection of tongue, skin, throat, eyes (red eyes, yellow eyes, etc.), feces, urine, external features (i.e., swelling), bleeding, body temperature.

Despite recent influences from Western medicine, ethnic people continue to rely on traditional medicine. This seems to confirm the therapeutic efficacy of local plants for the treatment of the most common and widespread pathologies [30].

### 2.3. Herbal Remedies

For medicinal preparations, roots, and rhizomes (23.61%), whole plant (22.88%), followed by bark and fruits (about 10% respectively) were used (Table 5).

The preference in selecting portions of plants could be related both to their availability during the year and to the higher concentration of active ingredients. For example, most informants considered the part of the stem closest to the ground or underground portions more effective from a therapeutic point of view. The preferential use of roots and rhizomes was also reported by Bhattarai [31] in its ethnobotanical survey carried out in Ilam District, Eastern Nepal. On the contrary, the study of Luitel et al. [26] in the Makwanpur District of Central Nepal found that the most frequent portions used for medicinal purposes were fruits/seeds, followed by whole plants and leaves.

**Figure 2:**
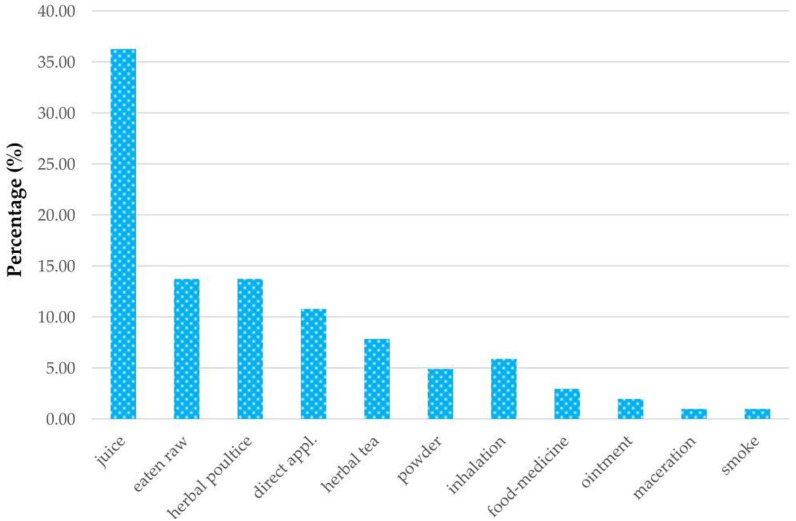
Histogram of the relative frequencies of the medicinal preparation forms used.

Considering only the 98 plant species used in human ailments, the informants reported various medicinal preparation forms. These were based on a single plant or were polyherbal formulations, fresh juice was the preferred form (36.27%) due to the simplicity of preparation by crushing the plant in a stone mortar and because this was also an excellent way of getting vitamins and minerals from the plant. The juice had to be taken within a short time after being prepared. Even raw plants (13.72%) were eaten for therapeutic purposes, especially roots, fruits, seeds, and tubers. Herbal poultices (13.72%) included “poultice”—generally prepared by crushing the plant portions to a pulpy mass - and “compresses” made of a piece of cloth soaked in the plant decoction or infusion. Other preparation forms were direct application (10.78%), herbal teas (7.84%), including decoction and infusion, inhalation (5.88%), powder (4.9%), vegetable used as food-medicine (2.94%), ointment (1.96%), maceration and smoke (0.98%, respectively) (Figure 2).

The informants generally preferred fresh plants because they found them more effective. Plant parts were generally prepared using hot or cold water as a solvent, but other solvents such as milk, honey, lemon juice and rapeseed oil were occasionally employed.

Single herbs, or multiple herbs combined to obtain original recipes, can be used. Particularly, a Newar medicinal recipe, used to treat various ailments, was found to be in practice. It is prepared with aerial parts of *Artemisia indica*, *Centella asiatica*, *Drymaria cordata*, *Mentha spicata*, *Oxalis corniculata* (Figure 1D), *Tagetes erecta*, seeds of *Oroxylum indicum*, sprouts dried of *Oryza sativa*, wood of *Picrasma quassioides* and limestone powder. A typical feature of Newari medicine is that different medicinal plants can be used to treat a number of diseases [32].

Herbal remedies for human ailments were administered through different routes: internal use (66.33%), topical application (23.47%), nasal application and others (10.2%).

The prescribed quantity was always approximate, depending on how the healer judged the severity of the disease. The frequency of taking the medicines has been described in terms of the number of times in a day and the duration as for number of days or weeks.

### 2.4. Data Analysis

To evaluate the ethnopharmacological importance of each plant species and the degree of agreement among informants some ethnobotanical indices were calculated. The informant consensus factor (Fic) was used to highlight medicinal plants of particular cultural relevance and the degree of agreement of informants about each category of ailments. The different ailments and diseases were classified into 14 categories (see Table 4) and a Fic value for each category was calculated. The results showed that fever category had the greatest agreement (0.49), followed by gastrointestinal and dermatological categories (0.29). Intermediate agreement among informants was recorded for metabolic, oral/dental/ENT (0.28) and musculoskeletal (0.21) categories, while the others had a Fic lower than 0.20 (Table 6).

The fidelity level (FL) was used to identify the most preferred species for treating certain ailments, showing that the most quoted species were *Calotropis gigantea* (100%) for dermatological diseases, *Drymaria cordata* (100%) against fever, *Mangifera indica* and *Wrightia arborea* (100%) for gastrointestinal disorders. For treatment of fever also *Oxalis corniculata* and *Centella asiatica* were reported, with a FL of 80% and 75%, respectively. Notably a FL of 75% was also found for *Curcuma caesia* for treatment of maternal ailments. To better underline the therapeutic properties of the most cited medicinal plants, we have reported some information on their main chemical compounds probably involved in the specific curative effect (Table 7), based on recent pharmacological investigations [33,34,35,36,37,38,39].

Plant species with highest relative frequency of citation (RFCs) were Achyranthes bidentata (0.28), Calotropis gigantea and Drymaria cordata (0.16), followed by Artemisia indica, Centella asiatica, Curcuma caesia, Daphne papyracea and Oxalis corniculata (0.13, respectively) (Table 8).

## 3. Discussion

Our results were in good accordance with other ethnobotanical studies carried out in Nepal and more in general, with the heritage of Traditional Asian Medicine. For example, the use of the latex from *Calotropis gigantea* for treatment of dermatological diseases (FL 100%) has been previously reported [23,40,41]. This plant is well-known in Ayurvedic medicine, reporting its many medicinal properties, including antimicrobial, antidiarrhoeal, antibacterial, antioxidant, anti-pyretic, and cytotoxic activities [42].

The antipyretic effects of *Drymaria cordata* (FL 100%), previously reported [43], can be associated with its content in saponins and related phytosterols, as well as phenols [34]. In addition, our informants have referred another medicinal use of plant juice drunken for the treatment of rhinitis and sinusitis. The same use of this plant has also been indicated in other areas of Nepal, but in this case the remedy administration was carried out by inhalation or fumigations [44,45].

*Mangifera indica* is a common tree in the home gardens of villages. Our informants described the use of the ripe mango peel to treat various gastrointestinal disorders (FL 100%). The use of this species for this category disease is confirmed by other studies on the folk medicine of Nepal [40], but bark and roots are generally the portions to which the greatest curative efficacy is attributed [23,46]. Therefore, the medicinal use of the fruit peel is typical of the survey area.

**Figure 3:**
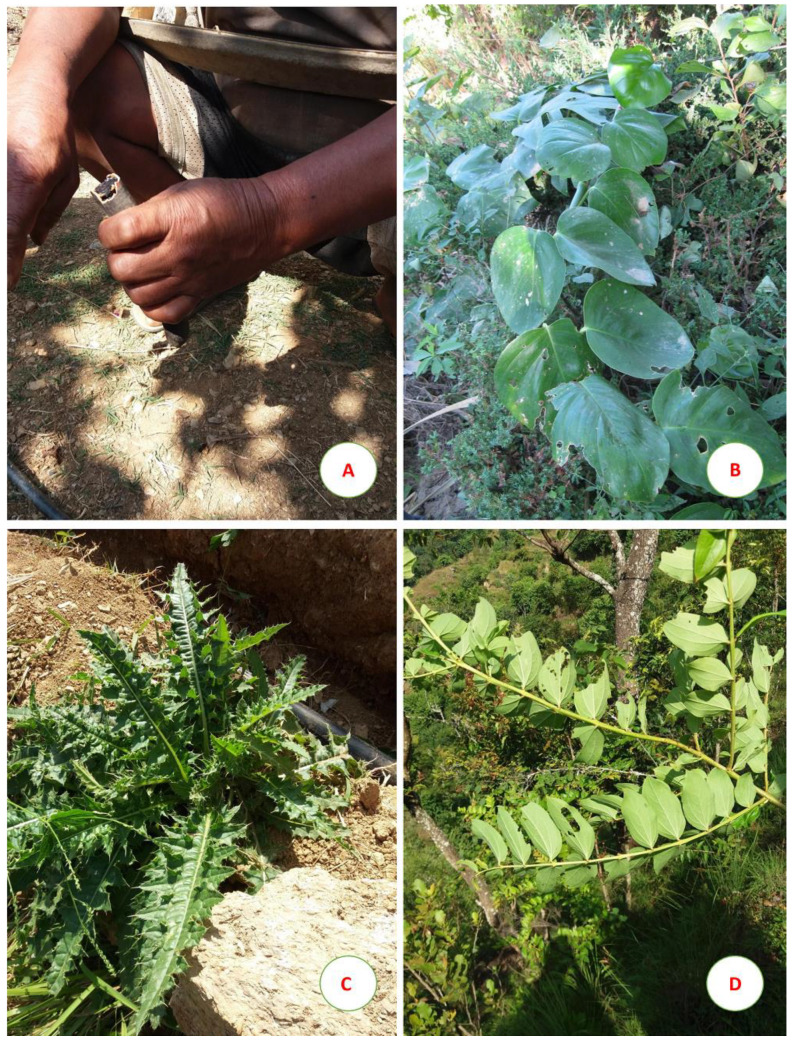
Informant with a pod of (**A**) Wrightia arborea; (**B**) Rhaphidophora glauca; (**C**) Cirsium wallichii; (**D**) Coriaria nepalensis.

Another plant species showing 100% FL indicated by our informants for gastrointestinal problems was *Wrightia arborea* (Figure 3A).

This plant, belonging to the Apocynaceae family, is widely used in Ayurveda, Siddha, and other traditional medicines to treat various human diseases [47]. The bark is used as an antidote against snake bites and scorpion stings, for curing menstrual and renal complaints and for its antipyretic and antibacterial activities. The root is used to bring relief in case of headache and fever, while the leaves as a diaphoretic, expectorant and to treat dysentery, toothache, and diarrhea [48,49]. In our survey, we found a previously unknown use of the *W. arborea* pod septum, consumed raw to cure intestinal disorders.

We also compared our data with those from previous studies conducted among ethnic communities living in hilly rural areas of the Kavrepalanchok District [16,17], and in neighboring districts of Central Nepal [19,20,23,24,25,26,27]. Such comparison (Table 2) revealed that 77 of the plant species (66%) had already been mentioned and that 44 of them (38%) had been indicated for similar uses in at least one case. Therefore, we have focused our attention on species or on unusual medicinal uses of plants that had not been referred previously.

Although mixing medicinal plants and minerals—like black salt, “chuna” (limestone powder) iron, copper, “silajeet” (*Asphaltum punjabianum*), alum, etc.—is a common practice among shamans and local healers (also for making “buti”, or amulets) [50], in our survey we found some original recipes. For example, pieces of copper were added to the bark of *Ficus semicordata* to make a preparation for treating bloody diarrhea. Another special combination of plants and mineral powder was used by Newar healers against many diseases such as fever, joint pain and others. These plants included aerial parts from *Centella asiatica*, *Tagetes patula*, *Mentha spicata, Oxalis corniculata*, *Drymaria cordata* and *Artemisia indica*, seeds of *Oroxylum indicum*, dry shoots of *Oryza sativa*, and wood of *Picrasma quassioides* Notably, *P. quassioides* is not present in any of the previous studies on the ethnobotany of the Tamang people. Its wood contains quassinoids with anticancer and antimalarial activity [51], other than a number of medicinal compounds with anthelmintic, antiamoebal, antiviral, bitter, hypotensive, and stomachic properties [52].

Another interesting use was reported for *Rhaphidophora glauca* (Figure 3B), an aroid liane native to the subtropical and warm temperate Himalayan regions. The juice obtained from the aerial parts was administered to women to improve pregnancy. Similar uses are reported for related Indian species such as *R. hookeri* whose stem juice was indicated for pregnant women [53], and *R. pertusa*, that showed luteolytic, oestrogenic, and follicle-stimulating activities in cattle and buffaloes [54]. Interestingly, none of the previous studies on Tamang folk medicine cited this specific use for this genus. On the contrary, the most common medicinal use of different *Rhaphidophora* species, i.e., the treatment of bone fractures in human and cattle [55,56], was not reported by our informants.

*Arenaria benthamii*, belonging to the Caryophyllaceae family, has been employed to fill pillows in case of cold and cough, and to reduce nasal mucus. Although the balsamic properties of this species and of other Caryophyllaceae, such as *Gypsophila arrostii* Guss. and *G. oldhamiana* Miq., are known [57], this route of administration had never been reported previously.

Another new finding concerns the use of root juice from *Strobilanthes pentastemonoides* for the treatment of high fever, while other studies only reported the use of this plant like fodder [58,59].

The stem juice obtained from branchlets of *Cipadessa baccifera* was highly regarded by shamans as a good remedy for counteracting food and drink poisoning. This use had never been previously reported, but a few studies have shown the effectiveness of this plant as an antidote against snake, scorpion, and insect bites [60,61].

Our informants reported that the root juice of *Cirsium wallichii* (Figure 3C) was useful for urinary problems, weakness, and malarial fever. Other studies cited the root of this species to treat gastric problems [62], while recently this species has been investigated also for antimicrobial and antioxidant properties shown by leaf, inflorescence, and bark extracts [63].

The use of the ripe fruits and plant juice of *Coriaria nepalensis* (Figure 3D) in case of indigestion is unexpected, because the leaves and fruits of many *Coriaria* species are considered poisonous in Asia, due to the presence of coriamyrtin with convulsive effects [64]. Nevertheless, in Chinese Traditional Medicine this species is used to treat various diseases [65], and the leaf juice was indicated as antiseptic among the Newar community of Kathmandu District [32].

**Figure 4:**
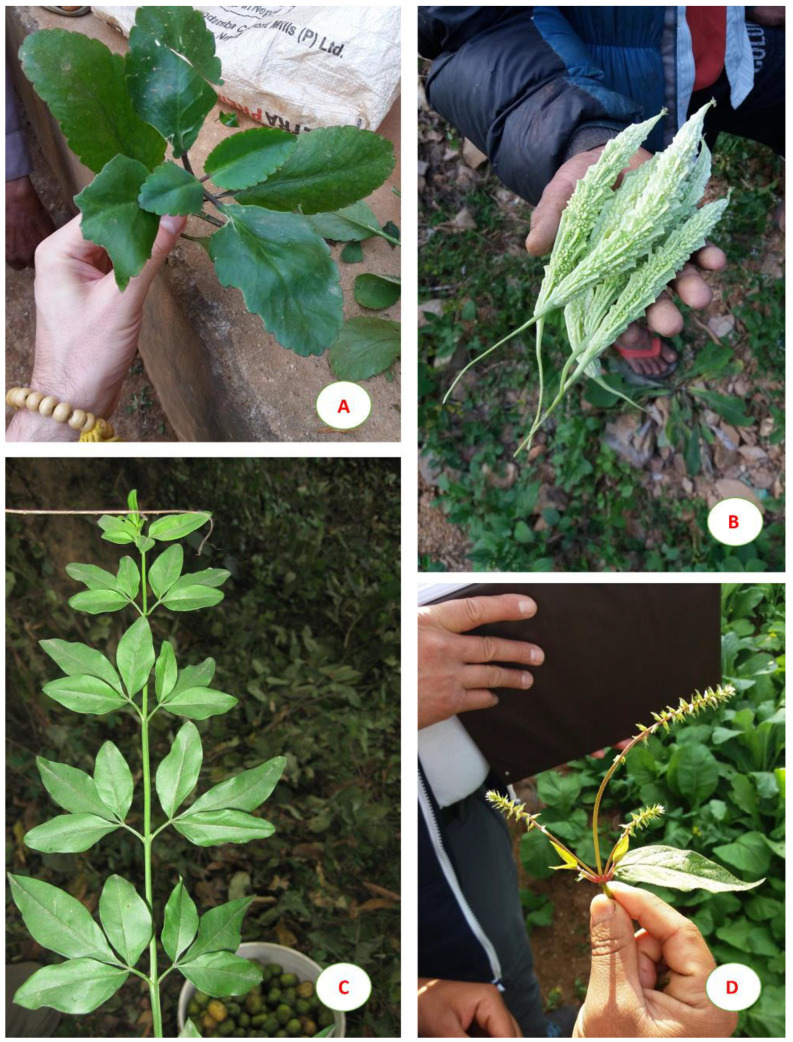
(**A**) Bryophyllum pinnatum; (**B**) Fruits of Momordica charantia; (**C**) Jasminum mesnyi; (**D**) Achyranthes bidentata.

The Crassulacea *Bryophyllum pinnatum* (Figure 4A) was described by our informants as useful against kidney stones, in agreement with other studies [66,67,68].

*Momordica charantia* (Figure 4B) is commonly used in the traditional medicine of different developing countries for its multiple properties, especially as antidiabetic [69]. In our study, this plant was mainly used by local people to lower blood pressure. Such effect, also reported in African folk medicine, was tested on normal and diabetic rats showing that an aqueous extract possesses hypoglycaemic and hypotensive properties [70].

*Jasminum mesnyi* (Figure 4C), commonly known as Japanese jasmine, has been reported in our survey to cure fever and typhoid. The antimicrobial potential of the leaf extract of this species has been recently demonstrated on Gram-positive and Gram-negative bacterial pathogens [71].

*Achyranthes bidentata*, (Figure 4D) belonging to Amaranthaceae, is a well-known medicinal plant very common near the villages and used by local people to treat various diseases such as fever (FL about 33%) and maternal ailments (FL about 44%). Accordingly, this species showed the highest relative frequency of citation. These findings are a novelty, because a previous study on the same area reported only the use of this species for the treatment of urinary ailments [16].

Finally, twelve of the 116 plants cited by our informants are also traded in the streets of the Kathmandu Valley for the preparation of popular medicinal remedies, being a valuable source of income for local people [72]: *Acorus calamus, Asparagus racemosus, Bergenia ciliata, Cautleya spicata, Curcuma angustifolia, Osyris wightiana, Phyllanthus emblica, Rubus ellipticus, Stephania glandulifera, Terminalia bellirica, Tinospora sinensis*, and *Valeriana jatamansi*.

Some informants (especially plant traders) referred that there is a great demand of *Asparagus racemosus* roots, because they are useful for stimulating milk production in buffaloes. For this reason, the collection and trade of this species should be controlled to prevent environmental depletion.

## 4. Materials and Methods

### 4.1. Study Area and Ethnic People

**Figure 5:**
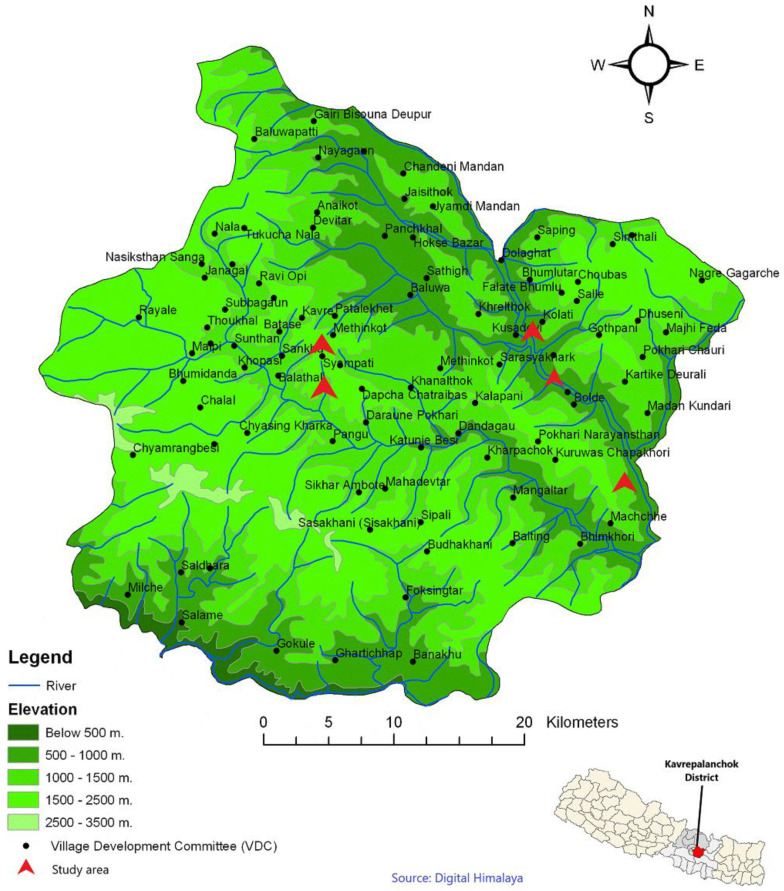
Map of the study area with the surveyed villages.

The Bagmati Pradesh (State) of Nepal comprises 13 districts, namely Sindhuli, Ramechhap, Dolakha, Sindhupalchok, Kavrepalanchok, Lalitpur, Bhaktapur, Kathmandu, Nuwakot, Rasuwa, Dhading, Makwanpur and Chitwan Districts. At the time of the 2011 Nepal census, Kavrepalanchok District had a population of 381,937. Of these, 50.9% spoke Nepali, 33.5% Tamang, 11.1% Newari, 1.6% Danuwar and 1.4% Magar as their first language [9]. The Kavrepalanchok District is located between 85°24’ to 85°49’ E and 27°22’ to 27°85’ N, with altitudes ranging from 275 (Dolalghat/Sunkoshi River) to 3018 m ASL (Bethanchowk hill). The total area is about 1396 km^2^ and the average temperature ranges from 10° to 31 °C [13]. This region has a subtropical climate and its vegetation is characterized by the forest of *Schima* sp., *Castanopsis* sp., *Pinus roxburghii* and *Alnus nepalensis* at the lower belt, while broad leaved oak forests of *Quercus* spp. are found at upper belt [73]. The Kavrepalanchok District consists of 13 Municipalities, out of which six are urban municipalities and seven are rural ones. The present survey was undertaken in six villages of the Temal Rural Municipality: Mukpatar (27°28’ N 85°48’ E), Maure (27°31’ N 85°45’ E), Chukha (27°32’ N 85°43’ E), Maure Bhanjyang (27°32’ N 85°44’ E), Lamagaun (27°33’ N 85°43’ E), Rackse Dhara (27°33’ N 85°43’ E), and in addition in the neighboring villages of Namo Buddha (27°34’ N 85°35’ E, Namobuddha Municipality) and Nyaupane Sanogon (27°33’ N 85°34’ E, Balthali Village Development Committee, VDC) (Figure 5).

This area is about 100 km far from Kathmandu and its villages are mainly occupied by the Tamang community. Although this rural area is not too far from the city centers, people living here are very deprived of basic infrastructures and facilities. Many people are illiterate and therefore the major portion of population is involved in agriculture, which is mostly subsistence farming. Some people are engaged in tourism industry as porters and guides, like drivers or other occupation. Since educational facilities for children are lacking and parents often consider education a trivial factor in life, children are not encouraged to get an education.

The criterion used in choosing the villages for this study was to have a sufficiently homogeneous survey area in terms of culture and land use. The informants belonged to the Tamang ethnic group (27 informants), and to other minor communities living in the study area, like Newar (2 informants) and Brahmin (3 informants). Tamang are one of the major Tibeto-Burmese speaking communities in Nepal, originating from Tibet. In Tibetan language “Ta” means horse and “Mang” means traders. So probably Tamang originally were horse traders. They account for 5.8% of the total people of the country [9] and currently most of them lives in the hilly regions of Nepal, adjoining sides of the Kathmandu valley.

In the study area the Tamang is the main ethnic group, while the other groups represent a minority. In the rural villages, small Newar and Brahmin family groups have been present for a few generations, with a consequent exchange of ethnobotanical traditions. For this reason, the proportional composition of our informants reflects this ethnic distribution.

Tamang people have a rich traditional knowledge linked to plants and animals, nevertheless only a few ethnobotanical studies concerned this culture [12,16,19,25,74,75]. According to the animistic vision of Tamang, the world is populated by numerous spirits living in nature and responsible for many events of life. Evil spirits, angry ghosts (*pichas*), and witches (*bokshi*) should be the cause of balance alterations in the human organism that cause diseases. The shamans (*jhankris*), acting as mediators between the spiritual and the material world, have developed techniques to identify the evil spirits, rituals to expel them, such as mantra (secret whisper) and amulets, and herbal preparations for humans and animals [76,77]. A few informants from the study area belonged to the Newar and Brahmin ethnic groups. The Newar are the indigenous inhabitants of the Kathmandu Valley and are known for their rich artistic and cultural tradition. Their main occupations are agriculture and plant trade [58]. Brahmin (*Bahuns* in Nepali Language) represent one of the Hindu Nepalese castes, and not a distinguished ethnic group. They live in the central area of Nepal, occupying fertile agricultural land at the foot of the Himalayan mountain range [78].

### 4.2. Field Survey and Data Collection

The present study was carried out in the months of October and November of 2017 and 2018, after the monsoon season. During two previous study trips (2015–2016) contacts were made with the local community in order to gather information on his daily habits, work, and relationship with the natural environment. Ethnobotanical and ethnomedicinal data on plants has been collected by interviewing 32 informants from the different villages of the study area.

For the choice of the typology and number of informants we have followed the “purposive sampling” approach according to Tongco [79], a nonrandom method, where the informants are selected by virtue of knowledge and experience. In our case, such key informants are the persons recognized by the community as depository of traditional knowledge about medicinal plants. This approach allows to collect a high amount of reliable data with a low number of interviews.

Key informants (24) were traditional healers selected by the following criteria: experience (local healers and shamans); age (knowledgeable elder villagers); occupation (farmers and plant traders).The interviews were conducted in Tamang and Nepali Language with the help of a bilingual speaking local guide. The data has been collected in compliance with the rules of the World Intellectual Property Organization (WIPO) related to traditional cultural expressions [80], and of the ethical guidelines of the international society of ethnobiology (ISE) [81]. The purposes and modalities of the research were carefully communicated to the informants and a prior informed consent (PIC) was obtained [82].

Different interviews and inquiry methods were followed, according to Martin [83] and Alexiades [84]: (a) informants were asked to freely list all plants of a certain ethnobotanical interest, with special attention to plants used for medicinal purposes (free listing); (b) healers and shamans were accompanied on their excursions in search of medicinal plants and followed during the healing ceremonies and herbal remedies preparation (walk-in-the-woods and participant observation). Some difficulties have been found in interviews with shamans because of the belief that revealing the medicinal properties of a plant deprives it of the therapeutic efficacy; (c) samples of plants were shown to the informants, asking them to identify species of ethnobotanical interest (specimen display); (d) small groups of people were interviewed about their ethnobotanical knowledge using the specimen display method (group interviews).

All information was collected through semi-structured questionnaires, including questions on the informants (gender, age, ethnic group, occupation, educational level, birth place, residence place, interview place, etc.), and the plants (local name, growth form, habitat, parts used, uses, preparation and routes of administration of herbal medicine). Plant specimens were collected and partly identified in the field with the help of local guides [58,85,86], and partly by one of us in the laboratory (RPC). The set of plant specimens was deposited in the Tribhuvan University Central Herbarium (TUCH), Nepal, with voucher numbers [87]. Plant nomenclature follows Plant of the World [88].

### 4.3. Quantitative Analysis

The collected data include plant species name, family, local name, altitude, location, voucher specimen number, parts used and ethnomedicinal uses. The data were then processed in ethnobotanical indices: Fic (informant consensus factor), FL (fidelity level), RFCs (relative frequency of citation), as followed.

#### 4.3.1. Informants Consensus Factor (Fic)

Informants consensus factor was calculated in order to find out the homogeneity in the information given by informants. This index is calculated for each ailment category with the following formula [89,90].

(1) $$Fic=\left(N_{ur}-N_{t}\right)/\left(N_{ur}-1\right)$$

where N_ur_ is the number of use reports in a particular illness category, and N_t_ the number of taxa or species used to treat that particular category. A high Fic value indicates the agreement among the informants on the use of taxa for a certain disease category.

#### 4.3.2. Fidelity Level (FL)

The fidelity level (FL) is the percentage of informants claiming the use of a certain plant for the same major purpose, and is calculated according to the following formula [84,91]:

(2) $$FL=\left(N_{p}/N\right)\times 100$$

where N_p_ is the number of informants that claim the use of a plant species to treat a particular disease, and N the number of informants that use the same plant as a medicine to treat any disease.

#### 4.3.3. Relative Frequency of Citation (RFCs)

This index is used to determine the local importance of each species in the study area. The formula used, according to Tardìo and Pardo-de-Santayana [92] is:

(3) RFCs=FCs/N

where FCs is the number of informants that cites the use of a plant species, and N the total number of informants.

## 5. Conclusions

The present study shows that the ethnic community, living in rural areas of the Kavrepalanchok District, still retains a rich traditional knowledge of medicinal plants which are an important source for primary health care. In fact, although some allopathic medicines are available in government “health posts”, most indigenous peoples rely on traditional local healers and shamans for their primary health care needs. Despite the close relationship with wilderness in this area, we have observed that this heritage is at risk. Young people are generally attracted to urban and Western lifestyles, and sometimes do not fully understand the value of traditional knowledge. Our data could be of help to the people of the rural municipalities of this area in order to plan bioconservation strategies. A recent example in the Temal region concerns an endemic species of handicraft interest, *Ziziphus budhensis*, which is cultivated and traded for Buddhist rosaries.

Similarly, the plants for which we have found novel therapeutic uses could be subjected to pharmacognostic investigation and cultivated in dismissed agricultural lands, turning them into a source of income and valorisation of the territory.

Therefore, the uses of medicinal plants in this area need to be explored and documented before oral traditions are lost forever. The active involvement of local populations in the valorisation, conservation, and management of medicinal plants will encourage future projects aimed at the sustainable development of the biological and cultural diversity of these rural areas of Nepal.

## Figures and Tables

**Table 1:** Distribution of informants gender and age.

Gender	Number of Informants by Gender and Class of Age (Years)					
	23–30	31–40	42–50	52–60	65–70	75 and >75
Men	2	7	4	3	7	3
Women	0	1	2	1	2	0
Total	2	8	6	4	9	3

**Table 2:** Plants used by ethnic people in Kavrepalanchok District, Central Nepal.

Family Scientific Name Voucher Number	Vernacular Name ^a^	Origin ^b^	Parts Used ^c^	Ethnobotanical Uses ^d^	Uses Description	Similar Uses References	Other Uses References
**Acanthaceae**							
*Justicia adhatoda* L. GA20171031NP011	Asuro (N) Basak (T)	W	L R	agr med/resp,fev,urogen med/fev	The leaves are used in horticulture as fertilizer. Dried leaves are smoked to cure cough. Powder of 4 dried leaves is mixed with honey (or water) and eaten, twice a day (morning and evening), to treat fever. In case of urinary problems: 3 dry leaves, after being left in water for 5 h, are cut into small pieces, honey is added, and the final product is eaten. Root juice is filtered and drunk 3 times a day to treat high fever.	[23,24,26,27]	Typhoid [23], rheumatic pain [24], sinusitis [25]
*Strobilanthes pentastemonoides* (Nees) T. Anderson (= *Goldfussia pentastemonoides* Nees) GA20181012NP052	Gathe (N)	W	R	med/fev	The root is cut into small pieces, pounded with water and the filtered juice is drunk 3 times a day to cure high fever.		
**Amaranthaceae**							
*Achyranthes bidentata* Blume GA20171113NP047	Dok, Datiwan (N), Ghyurupuchu, Ghiughiuru (T)	W	R Wp	med/fev med/mat med/urogen vet med/met,fev,derm	Root is crushed with water and the filtrate is drunk 3 times per a to cure fever and typhoid. One day of treatment is often sufficient for healing. Root juice is drunk 2 times day to promote childbirth. The juice obtained by squeezing the root is drunk to treat urinary problems (blood in the urine). Root juice stimulates lactation in buffaloes. Plant juice is drunk for blood purification and to cure fever. The well-washed plant is rubbed directly on the skin, to treat pimples, boils, itchy pustules.	[16]	Asthma [16], gastric problems, toothache [17]
*Amaranthus cruentus* L. GA20171030NP002	Latte (N)	C	L/Sd	food	The leaves are cooked like spinach, added to soups, or eaten raw. The seeds are cooked and used for soups (*satoo*), or ground to obtain a flour for bread (*chapati*).		
*Amaranthus hybridus* L. GA20171030NP003	Latte (N)	C	L/Sd	food	The leaves are cooked like spinach, added to soups, or eaten raw. The seeds are cooked and used for soups (*satoo*), or ground to obtain a flour for bread (*chapati*).		
*Amaranthus viridis* L. GA20181016NP098	Gaute bangan (T)	W	R	med/fev	Root juice is used to cure high fever.		
**Anacardiaceae**							
*Choerospondias axillaris* (Roxb.) B. L. Burtt & A. W. Hill GA20171113NP048	Lapsi (N)	C	Fr	food	Fruits are eaten fresh or pickled.		
*Mangifera indica* L. GA20181017NP104	Aanp (N)	C	Fr	med/gast	The peel of fruits is boiled and eaten to cure gastritis.	[16,23,25]	Jaundice,rheumatism [16],dysentery [23]
*Searsia parviflora* (Roxb.) F. A. Barkley (= *Rhus parviflora* Roxb.) GA20181016NP093	Satibro (T)	W	Fr	med/gast	Ripe fruits are eaten to treat stomach problems and diarrhea.		
**Apiaceae**							
*Centella asiatica* (L.) Urb. GA20181018NP111	Tajoimra (T), Kholachagaian, Kolacha (Ne)	W	Wp R	med/fev,ant,musc,nerv med/urogen	Plant juice is drunk to cure fever and sometimes is mixed with the juice of *Drymaria cordata* to treat high fever. A typical recipe (*) is used to cure fever, internal fever, food poisoning, joint pain, migraine. Root juice is drunk to cure urinary problems.	[19,23,26,27]	Blood purification [19], indigestion [20], skin diseases [24], sinusitis [25]
**Apocynaceae**							
*Calotropis gigantea* (L.) Dryand. GA20171030NP004	Arka (sanskrit), Aank, Akh (T)	W	Lx	med/derm,musc	Latex is used directly on the skin to stop bleeding wounds and to treat ankle sprains.		Joint fracture [23]
*Catharanthus roseus* (L.) G. Don GA20171101NP017	Barhamase phul (N)	N	Wp	dom	The plant is used as an ornamental in home gardens.		
*Wrightia arborea* (Dennst.) Mabb. GA20181017NP108	Glemindhu (T)	W	Fr/Sd	med/gast,fev	The soft septum between one seed and the other, is eaten to cure flatulence, intestinal pain with dark stools, insolation with fever.		
**Araceae**							
*Acorus calamus* L. GA20181016NP096	Seda (T)	C	Wp	med/derm	The plant is grated on a stone and applied on skin wounds.		Cough/cold [19], roundworm, hookworm [23], fever [26]
*Colocasia esculenta* (L.) Schott GA20181015NP087	Pidalu (N), Taia (T)	C	Rh	med/gast food	Some pieces of boiled rhizome are eaten to cure the constipation of children (1–5 years). Boiled rhizome is commonly eaten as food by the people, especially for breakfast.		
*Rhaphidophora glauca* (Wall.) Schott GA20171111NP045	Birlahra (T)	W	Wp	med/mat	To promote pregnancy, the juice of the plant is taken 3 times a week: Saturday,Tuesday,Thursday.		
**Asparagaceae**							
*Agave cantala* (Haw.) Roxb. ex Salm-Dyck GA20171030NP001	Ketuki (N)	N	L	handicr	Dried leaves are used for roofing. Leaves provide fibers used to make ropes, cordage, and twine.		Diuretic, anti-syphilitic [20], worms on wound [27]
*Asparagus racemosus* Willd. GA20181015NP084	Kurilo (N)	C	R	med/met vet	Root juice is drunk to cure jaundice. Root juice is mixed with corn flour and given to the buffaloes for about 2–3 days, twice a day, to stimulate milk production.	[19,23]	Urinary disorders, stimulation of milk production in women [19]
**Asteraceae**							
*Ageratina adenophora* (Spreng.) R. M. King & H. Rob. GA20171109NP023	Banmara (N)	W	L	med/derm	Five to seven leaves are crushed with water and the green liquid extracted from the obtained paste is applied on cuts and wounds to stop the bleeding.	[16,17,19,25,26,27]	Fever, eyes insomnia [16]
*Artemisia indica* Willd. GA20171109NP025	Titepati (N), Dusun (Ne)	W	Wp Ys	rel med/ant,resp,fev,musc,nerv med/resp	The dried plant is used as incense. Plant juice is drunk to treat food poisoning and cough. A typical recipe (*) is used to cure fever, internal fever, food poisoning, joint pain, migraine. 4–5 shoots are crushed with water, the juice is filtered and drunk for the treatment of throat irritations.	[17]	Gastritis, paralysis, [23], rheumatic pain [24], cuts, scabies [25], louse, worms [27]
*Bidens pilosa* L. GA20181018NP110	Buk tinai (T)	N	Fl	med/fev,nerv	The juice of flowers is drunk, twice a day (morning and evening), to cure fever and migraine.		Cuts and wounds [16]
*Blainvillea acmella* (L.) Philipson (= *Spilanthes acmella* (L.) L.) GA20181012NP054	Saprumo (T)	N	R	med/resp	Root juice is drunk twice a day (morning and evening) to cure cough and cold.		
*Blumea aromatica* DC. GA20181015NP085	-	N	Fl	food	The dried flowers are mixed with *Clematis buchananiana* and *Oryza sativa*, and fermented for a week to obtain a popular alcoholic drink (*chhaang*).		
*Cirsium wallichii* DC. GA20181012NP061	Thakal (N), Achangpolo (T), Chwacan (Ne)	W	R	med/urogen,genh,fev	Root juice is drunk to treat urinary problems (blood in the urine), weakness (the juice is taken twice a day), malarial fever.		
*Duhaldea cappa* (Buch.-Ham. ex D. Don) Pruski & Anderb. (= *Inula cappa* (Buch. Ham. ex D. Don) DC.) GA20181013NP069	Ranaven, Ranabhyang (T)	W	R	med/fev	Root juice is drunk to cure fever.		Snake bite, menstrual disorders, epilepsy [19], headache [23], gastritis, indigestion [24]
*Eclipta prostrata* (L.) L. GA20181013NP066	Mashi mra (T)	W	St/L	med/derm	The hand-crushed plant is applied directly to the wounds of the skin.		
*Elephantopus scaber* L. GA20181013NP067	Buti jhar (N), Tinai (T)	W	R Wp	med/ant med/met	Root juice is drunk once/twice times in the nighttime, in case of food poisoning. Plant juice is taken to cure jaundice. In case of infant jaundice, the mother drinks the juice before breastfeeding the sick child.		Headache, sinusitis [23]
*Galinsoga parviflora* Cav. GA20171113NP049	Bhuitimur (N)	N	Fl	med/ENT	3 flowers are put on the sore tooth for 15–30 min. Once is often enough to relieve toothache, but the treatment can be repeated a second time after 3 days.		
*Smallanthus sonchifolius* (Poepp.) H. Rob. GA20171113NP051	Bhuishyau (N)	C	R	med/met food	The tuberous root, cleaned and eaten raw, once per week, is useful in case of diabetes. Tubers are slightly sweet, crispy and juicy and are usually eaten raw like a fruit.		
*Tagetes erecta* L. (= *Tagetes patula* L.) GA20171030NP005	Sayapatri (N), Saipatri sun (Ne)	C	Fl	rel med/derm,fev,ant,musc,nerv	Flowers are used for the creation of garlands and decorations for weddings, festivals, and other religious events. Flowers are rubbed on the injured and swollen skin. A typical recipe (*) is used to cure fever, internal fever, food poisoning, joint pain, migraine.		
**Berberidaceae**							
*Berberis asiatica* Roxb. ex DC. GA20181012NP059	Chutro (N)	W	R	med/fev,ENT,gast	The root is boiled for 5 minutes in about 1/2 L of water and the infusion is drunk to treat fever, toothache, “red eyes” (conjunctivitis), mouth infections, diarrhea.	[19,20,25]	Blood purification [19]
**Bignoniaceae**							
*Oroxylum indicum* (L.) Kurz GA20181018NP113	Tatelo (N), Tarlason (Ne)	W	Sd	rel med/derm fev,ant,musc,nerv	Seeds are widely used in religious ceremonies and divinatory practices of shamans. Seed are applied externally to heal cuts and wounds. A typical recipe (*) is used to cure fever, internal fever, food poisoning, joint pain, migraine.	[17,26,27]	Dysentery [26], jaundice [27]
**Brassicaceae**							
*Brassica rapa* L. (= *Brassica campestris* var. *sarson* Prain) GA20171031NP006	Sarson (N)	C	Sd	food med/ENT med/musc	The oil obtained by seeds is used to cook. The ointment obtained mixing hot rapeseed oil and dried bark of *Myrica esculenta,* is applied externally to treat earache. The ointment obtained mixing hot rapeseed oil with leaves of *Abrus precatorius,* is used for massages in case of joint pain, twice a day.	[16]	
**Capparaceae**							
*Crateva religiosa* G. Forst. GA20181015NP088	Siplekan (N)	W	Fr	med/derm,ENT	Dried fruits are grated on a stone with the addition of water and the mixture is applied to the wounds of the skin. The fresh fruit liquid is applied externally to treat the swelling of the dental glands and the affected part is bandaged with “Nepali paper”.		
			L	med/gast	The young leaves are boiled and eaten to treat stomach pain.		
**Caprifoliaceae**							
*Valeriana hardwickii* Wall. GA20171112NP046	Daling (T)	W	R	med/gast	Fresh root juice or infusion of the dried root, is taken twice a day for a week, to cure gastric problems and vomiting.		
*Valeriana jatamansi* Jones ex Roxb GA20181015NP082	Jatamasi (N), Dhalin (T)	W	R	med/gast,nerv	Root juice is taken to treat diarrhea in children. With the dried roots shamans produce the incense for the treatment of anxiety and insomnia.		Fire burns [26]
				rel	Incense from the plant root is used for religious purposes.		
**Caricaceae**							
*Carica papaya* L. GA20171031NP007	Papaya (I)	C	Fr	food	Plant is cultivated in kitchen gardens for its edible fruits.		
**Caryophillaceae**							
*Arenaria benthamii* Fenzl ex Torr. and A. Gray GA20181012NP057	Tangne (T)	W	Wp	med/resp	The pillows are filled with the plant so that the active ingredients are inhaled during the night to cure fever and breathing problems.		
*Drymaria cordata* (L.) Willd. ex Schult. GA20171111NP042	Abijalo (N),Tangar, Abijal (T), Abisal ghe, Kai bugain (Ne)	W	Wp	med/fev,gast,ant,resp,musc,nerv	The plant parts are pounded, boiled in water for about 5 min and the filtrate is drunk once a day (morning or evening) for 3 days, to cure fever. Plant juice is drunk twice a day to cure fever and stomach infections. Plant juice is drunk to treat food poisoning, rhinitis, and sinusitis. Sometimes the plant juice is mixed with that of *Centella asiatica* to cure high fever.	[24,25]	
					A typical recipe (*) is used to cure fever, internal fever, food poisoning, joint pain, migraine.		
**Combretaceae**							
*Terminalia bellirica* (Gaertn.) Roxb. GA20181017NP107	Barla (T)	W	Fr	med/resp,gast	Fruits are kept in the mouth and sucked like candy to cure cough and gastric problems.	[20,23,26,27]	Cold, cough [27]
**Convolvulaceae**							
*Cuscuta reflexa* Roxb. GA20181013NP065	Sikari lahara (N) Sky grass (english)	W	St	med/met	Used in case of jaundice: (a) the plant juice is drunk; (b) the plant is pounded, boiled in water for 2–3 min and the filtrate is drunk.	[19,20,23,24,25,26,27]	Bone fractures, body swelling [19], roundworm, depression [23]
**Coriaraceae**							
*Coriaria nepalensis* Wall. GA20181017NP101	Bhujinshin (N) Hakupaku (T)	W	Fr L/St	med/gast	Ripe fruits and plant juice are taken to treat indigestion.		
**Crassulaceae**							
*Bryophyllum pinnatum* (Lam.) Oken GA20181012NP060	Kidney stone medicine	N	L	med/urogen	Leaf juice is drunk, or the leaves are eaten raw to cure urinary problems.		
**Cucurbitaceae**							
*Momordica charantia* L. GA20181016NP090	Karela (N)	C	Fr	med/card food	Fruits are consumed to control high blood pressure. Unripe fruits are cooked as a vegetable or pickled.		
**Dioscoreaceae**							
*Dioscorea bulbifera* L. GA20171110NP032	Dhingyui mindhu (T)	W	R	med/mat	The root (white color) is cut into small pieces and crushed to obtain a red juice, taken once a day regularly, by women with menstrual cycle disorders, for preventive purposes.		Piles, dysentery, syphilis, ulcers [16], pneumonia [19]
**Euphorbiaceae**							
*Euphorbia hirta* L. GA20181017NP103	Rato lahare (N) Walagughi (T)	W	Lx	med/derm,musc	Latex is applied on skin wounds and joint trauma without bone fractures.	[16,26]	Diarrhoea/dysentery, respiratory diseases, snake bites [16], excessive menstrual flow [24]
*Jatropha curcas* L. GA20171031NP010	Arin,Sajiwan (N) Mandhar (T)	N	St Fr/Lx	med/ENT med/derm	The most tender twigs are used for cleaning the teeth. Dried fruits or latex are applied externally in case of skin infections.	[23,24,26]	Gum problems [24]
*Ricinus communis* L. GA20181018NP116	Taturoro (Ne)	N	Fl	vet	The flowers are pounded with water and the paste obtained is applied to treat skin problems of cattle.		Bone fractures [23], worms in the teeth [24]
**Fabaceae**							
*Abrus precatorius* L. GA20181012NP055	Rati geri (N)	W	R L	med/nerv,resp,ant med/musc	The root is grated on a stone and inhaled to treat migraine. The root is grated on a stone, mixed with honey, and taken to cure cough. The dried root is pulverized and applied on snake bites, and the affected part is banded with “Nepali paper”. The ointment obtained by cooking the leaves with rapeseed oil is used to massage the aching joints, twice a day.		Stomach problems [27]
*Albizia julibrissin* Durazz. GA20181016NP097	Shirish (N)	W	R	med/musc	The root is cut into small pieces and boiled for more than 3 h along with *Osyris wightiana* and *Senegalia catechu*; the filtered juice is applied externally in case of bone fractures and the affected part is bandaged with “Nepali paper”.		
*Bauhinia variegata* L. GA20181012NP058	Koiralo (N)	W	Br	med/ENT,met,ant	The bark is boiled in about half liter of water. So water is used for gargling, in case of mouth infections and toothache. Even the dried bark can be used to treat toothache. The dried bark is powdered, mixed with *Zingiber officinale* powder and water. The dough obtained is applied externally on the throat, twice a day (morning and evening) in the treatment of goiter. Dried and pulverized bark is mixed with the juice of *Citrus × limon*; the dough obtained is applied on snake bites and the part is bandaged.		Diarrhoea/dysentery, piles [20], gastritis [25], fever [27]
*Lablab purpureus* (L.) Sweet GA20171110NP033	Lahare guki (T)	C	L	med/gast	The leaves are boiled for about 5–10 minutes, resulting in a *daal*-like soup, which is consumed regularly to treat *kabjiat* (constipation).		Ringworm on skin [27]
*Phaseolus vulgaris* L. GA20171031NP014	Rajama (N)	C	Fr/Sd	food	Fruits and seeds are used as vegetable and for cooking *daal* (lentil soup).		
*Saraca asoca* (Roxb.) J.J.de Wilde GA20181017NP106	Ashoka (N)	W	Sd	med/musc	The seeds are taken with water to bring relief in case of bone fractures.		
*Senegalia catechu* (L. f.) P.J.H.Hurter and Mabb. (=*Acacia catechu* (L. f.) Willd.) GA20181016NP095	Khayar (T)	W	R	med/musc	The root is cut into small pieces and boiled: the decoction is applied externally to promote the healing of bone fractures. Another recipe: small pieces of root are boiled for more than 3 h along with *Osyris wightiana* and *Albizia julibrissin*; the liquid obtained is applied on the affected part and the part is banded with “Nepali paper”.	[26]	Diarrhoea/dysentery [23], fever [26]
**Gentianaceae**							
*Swertia angustifolia* Buch.-Ham. ex D. Don GA20181014NP079	Chiraito (N), Kampman (T)	W	R/L Wp	med/fev	To treat fever: (a) root and leaf juice is drunk; (b) the whole plant is put in water for 12 h, then the macerate is drunk twice a day.	[16]	Blood purification, bile diseases, cough/cold [16]
**Iridaceae**							
*Iris domestica* (L.) Goldblatt & Mabb. GA20171110NP029	Darware mindhu (T)	C	R	med/gast,ant	The root juice is used in case of gastric problems and poisoning, once a day, in the morning.		Diarrhoea [27]
**Lamiaceae**							
*Colebrookea oppositifolia* Sm. GA20171031NP008	Dhursil (N), Busul sul (T)	W	Fl St L	rel med/derm med/gast	Flowers are sold in urban markets for temple offerings. Thin stem filaments are applied to cuts and wounds and the affected part is bandaged. Hand-crushed leaf juice is drunk by children in case of liquid diarrhea accompanied by abdominal pains.	[16,17]	Epilepsy, fever, headache, sinusitis [16], arthritis [17]
*Leucas cephalotes* (Roth) Spreng. GA20181013NP070	Topdoi mra (T)	W	Fl	med/met,musc	The infusion of flowers is drunk to cure jaundice and joint pain.		
*Mentha spicata* L. GA20181018NP112	Naasun (Ne)	W	Fl	med/fev,ant,musc,nerv	A typical recipe (*) is used to cure fever, internal fever, food poisoning, joint pain, migraine.		Gastric and intestinal disorders [16], insomnia [25]
*Ocimum tenuiflorum* L. GA20171031NP012	Tulsi (N)	C	Wp	rel med/gast,ENT	The plant is considered sacred and is used in the worship of Vishnu. The plant is used to prepare a herbal tea useful for gastric problems and dry mouth.		Blood pressure control, ear pain, respiratory diseases, typhoid [23]
*Perilla frutescens* (L.) Britton GA20171031NP013	Silam (N)	W	Sd/L	food	Toasted seeds are used to prepare a spicy sauce (*silam ko achar*); the leaves cooked like spinach; dried leaves are used to prepare an healthy herbal tea.		
*Pogostemon benghalensis* (Burm. f.) Kuntze GA20171111NP044	Rutula (T)	W	Wp	med/resp	Plant juice is filtered and drunk, twice a day for a week, to treat dry and tickly cough.	[23,27]	Typhoid [23]
**Lauraceae**							
*Cinnamomum glanduliferum* (Wall.) Meisn. GA20181016NP099	Tagba (T)	W	R	med/musc	The root is crushed, boiled for more than an hour and the mush obtained is used to massage the painful joints, twice a day.		Toothache, wounds [19]
*Litsea cubeba* (Lour.) Pers. GA20181013NP071	Siltimur (N)	W	Fr	med/gast	The dried ripe fruits, pulverized and dissolved in water, are taken twice a day to treat stomach problems.		Cholera [20]
*Machilus odoratissima* Nees (= *Persea odoratissima* (Nees) Kosterm. GA20181014NP076	Kaulo (N)	W	Br	med/card,musc,urigen	Dried bark is cut into small pieces, reduced to powder, mixed with honey and taken 7 times a day. Useful for heart attack, bone fractures, poor blood circulation, urinary problems.		
**Lythraceae**							
*Woodfordia fruticosa* (L.) Kurz GA20171110NP038	Daduimre, Bhyur ghara (T)	W	R Ys	med/gast	Root juice is drunk for stomach problems. The juice of about 1 kg of twig young shoots (red color) is drunk once/twice times a day to treat abdominal pain with blood in the stool.	[20,23,25,26,27]	
**Malvaceae**							
*Gossypium arboreum* L. GA20171031NP009	Kopi (T)	C	Sd	handicr	The fibres from the seeds are used in the production of blankets and wicks for incense.		
**Melastomataceae**							
*Osbeckia nepalensis* Hook. GA20181014NP075	Chulsi (N)	W	Wp	med/derm	The mixture obtained by crushing and mixing the plant with *Rubus ellipticus* whole plant, is applied directly on skin infections that tend to expand, especially on the abdomen.	[24]	Fever [27]
**Meliaceae**							
*Cipadessa baccifera* (Roth) Miq. GA20181016NP100	Painati (T)	W	Br	med/ant	The juice from some stem slices is drunk to cause vomiting against the food/drink poisoning.		Cough/cold [16]
*Melia azedarach* L. GA20181017NP105	Bakaino, Bakena (N)	W	Br	med/nerv,resp,fev,gast	The bark powder, mixed with honey, is consumed to treat migraine. A rag, soaked in the boiling water of the bark, is put on the forehead to treat cooling diseases. The bark is cut into small pieces, reduced to powder, mixed with *Citrus × limon* juice and honey, and eaten to cure fever. The bark juice is drunk twice a day for 3 days in case of gastric infections.	[16,20,26]	Skin diseases, hysteria, rheumatic pain [16], diarrhoea, constpation, cholera [23]
*Toona hexandra* (Wall.) M. Roem. (= *Toona ciliata* M. Roem.) GA20171101NP022	Tuni (T)	W	Wood	handicr	Wood is used for the production of furniture.	[16,17]	Infantile dysentery, ulcer and boils [16]
**Menispermaceae**							
*Stephania glandulifera* Miers GA20171110NP037	Gundri gano (T)	W	R	med/gast,mat,ant	Root juice is drunk once a day (morning or evening) to treat gastritis, menstrual disorders, and food poisoning.		Cough [27]
*Tinospora sinensis* (Lour.) Merr. GA20181015NP081	Gurjo (N)	W	St	med/genh,card	Stem juice or pieces of it are taken in case of cancer and piles.		Menstruation problems [20], diarrhoea, dysentery, stomachache [23]
**Moraceae**							
*Ficus racemosa* L. GA20181013NP068	Dumri (N)	W	Fr Lx	med/card med/derm	Ripe fruits are eaten to treat blood circulation disorders. Latex is applied to the skin affected by dermatological diseases.	[27]	Diarrhoea [26]
*Ficus semicordata* Buch.-Ham. ex Sm. GA20171110NP034	Ngedhore (T)	W	Br	med/gast	The bark of stem portion near the ground is crushed and boiled in a copper pot for about 3 h, adding small pieces of copper. The filtrate is drunk adding honey, twice a day, to treat dysentery with blood in the stool.		Scabies [25], wounds [26]
**Musaceae**							
*Musa* x *paradisiaca* L. GA20171101NP020	Kera (N)	C	Fr	food	Ripe fruits are edible and green bananas are cooked like vegetables.	[16,26]	Intestinal disorders, diabetes, uremia, nephritis, gout, hypertension, cardiac diseases [16], jaundice [27]
**Myricaceae**							
*Myrica esculenta* Buch.-Ham. ex D. Don GA20181014NP072	Kaphal (N)	W	Br	med/nerv,ENT,derm,resp	Dry bark powder is used in various remedies: it is inhaled 3 times a day to treat headache; in case of toothache, the teeth are washed with the mixture of bark powder and *Citrus × limon*; to treat earache, the bark powder is mixed with hot rapeseed oil to make an ointment for earache; to treat skin problems, the bark powder is applied to the skin with a “Nepali paper” bandage. For the treatment of sore throat the fresh bark is cut into small pieces which are placed inside the leaves of *Piper betle* (“paan”) and eaten like candy.	[19,27]	Constipation [17], diarrhoea, asthma [19], bronchitis [24], cholera [26]
**Myrtaceae**							
*Psidium guajava* L. GA20181016NP092	Amba (T), Guava (I)	N	Br	med/gast	The bark juice from the stem portion near the ground is drunk to treat severe belly pains with blood in the stool.	[16,20,23,26,27]	Skin problems, rheumatism, cholera, headache [16], anthelmintic [23],blood pressure control [25]
*Syzygium cumini* (L.) Skeels GA20181015NP080	Jamuna (N)	W	Fr	med/met	The dried ripe fruit powder is diluited with water and drunk twice a day to control diabetes.		Diarrhoea [23], typhoid [26]
**Oleaceae**							
*Jasminum mesnyi* Hance GA20171113NP050	Ghyi fui (N), Gaiful (T)	N	R/Wp	med/fev	Root juice is taken once a day for 3 days to cure fever and the juice of the whole plant is used to cure typhoid.		
*Nyctanthes arbor-tristis* L. GA20181014NP074	Parijat (N)	W	L	med/fev	Leaf juice is drunk to cure fever.		Cold/cough [26,27]
**Oxalidaceae**							
*Oxalis corniculata* L. GA20181018NP114	Pang qui, Nakbruigumba (T), Pauja gai (Ne)	W	Wp	med/musc,fev,ant,nerv	Plant juice is drunk to treat joint pain and internal fever. A typical recipe (*) is used to cure fever, internal fever, food poisoning, joint pain, migraine.	[23,26]	Cataract [24], sinusitis [25], conjunctivitis, typhoid [26]
**Phyllanthaceae**							
*Phyllanthus emblica* L. GA20171101NP021	Amala (N)	W	Fr	med/resp	The fruits are consumed as expectorants in case of cough and sore throat.	[23,26,27]	Hearth pain, constipation, diarrhoea [23], gastritis [26]
**Piperaceae**							
*Piper betle* L. GA20181014NP077	Paan (N)	W	L	med/resp	The leaves of *Piper betle* (“paan”) are used, in case of sore throat, to envelop small pieces of bark of *Myrica esculenta*, and therefore taken like candy.		
*Piper retrofractum* Vahl GA20171110NP036	Pan gughi (T)	N	St	med/gast	The stem, fresh or dried, is used for the treatment of gastric disorders. The stem is chewed or pounded to obtain a juice to drink.		
**Plumbaginaceae**							
*Plumbago zeylanica* L. GA20171111NP043	Chitu (N), Ping chittu (T)	W	Wp L	med/gast food	Plant juice is drunk, twice a day for a week, to treat gastric disorders. Young leaves are cooked in rapeseed oil.	[23]	Skin diseases [23], retention of urine [26]
**Poaceae**							
*Eulaliopsis binata* (Retz.) C.E.Hubb. GA20171101NP018	Arkhen khar (N)	W	Wp	dom	The dried plant is used as thatching roof.	[16]	
*Oryza sativa* L. GA20181016NP091	Sun (T), Nalasun (Ne)	C	Fr Ys	food med/fev,ant,musc,nerv	Rice is used in the preparation, along with *Blumea aromatica* and *Clematis buchananiana,* of the “chhaang” alcoholic beverage. A typical Newar recipe (*) is used to cure fever, internal fever, food poisoning, joint pain, migraine.	[16]	Hearth inflammation, indigestion [16]
*Saccharum officinarum* L. GA20171109NP026	Ukhu (N)	C	St	food med/urigen	Stem is sucked like candy or crushed to extract the sweetened juice. The stem, preferably the portion closest to the ground, is chewed when the bladder feels swollen and the urine is dark yellow.	[16]	Jaundice, stomach disorders, skin ulcers, seminal weakness [16]
*Zea mays* L. GA20181016NP094	Makai (N)	C	Fr	vet	Corn flour is mixed with *Asparagus racemosus* root juice and the mixture is given to buffaloes for about 2–3 days to stimulate milk production.		
**Polypodiaceae**							
*Nephrolepis cordifolia* (L.) C. Presl GA20181014NP073	Tui amala (T)	W	R	food	Watery root tubers are eaten as a snack to reduce thirst.	[24]	Bone fractures [26]
**Pteridaceae**							
*Hemionitis anceps* (Blanf.) Christenh. (=*Cheilanthes anceps* Blanf.) GA20171111NP040	Rani sinka (T)	W	St/L	med/gast	The plant is cleaned, boiled for about 10 min and consumed 3 times a day (morning, afternoon, evening) for 5–6 days, 10 days maximum,in case of stomach problems.		
**Ranunculaceae**							
*Clematis buchananiana* DC. GA20181015NP086	Chyanmangre (T)	W	St/R Wp	med/resp,gast food	The plant is crushed, wrapped in a cloth, and inhaled to treat sinusitis and allergic rhinitis. Root juice is drunk to cure gastric problems. The plant mixed with *Blumea aromatica* and *Oryza sativa*, is left to ferment for a week, obtaining a popular alcoholic beverage, “chhaang”.		
**Rosaceae**							
*Rubus ellipticus* Sm. GA20181014NP078	Aniselu (N), Polang (T)	W	R Wp	med/resp,gast med/derm	The hand-crushed root is inhaled to treat rhinitis and sinusitis. The root juice is taken for gastric problems. The crushed plant, mixed with *Osbeckia nepalensis*, is applied on skin infections.	[19,24,25,26,27]	Typhoid [19], fever [24,25]
**Rutaceae**							
*Aegle marmelos* (L.) Corrêa GA20181012NP056	Bel (N)	W	Fr	food med/gast,fev	Ripe fruits are consumed or mixed with cold water to prepare a refreshing drink (*sarbat*). Ripe fruits are consumed to treat diarrhoea and fever.	[20,23,26]	Scabies and roundworm [23], diabetes [24]
*Boenninghausenia albiflora* (Hook.) Rchb. ex Meisn. GA20171111NP039	Thangkap mra (T)	W	Wp	med/fev med/nerv	Plant juice or boiled plant is taken 2–3 times a day (preferably in the morning) in the treatment of fever, until remission of symptoms. In case of headache, the crushed plant is applied on the forehead, inhaled, or fumigated.		Cold, insect repellent [27]
*Citrus × limon* (L.) Osbeck GA20181013NP062	Nibuwa (T)	C	Fr	food med/ant,ENT,fev	It is used as a flavoring. The *Citrus × limon* juice, mixed with the dry and pulverized bark of *Bauhinia variegata,* is applied on the snake bites and the part is bandaged. Lemon juice, mixed with the dry and pulverized bark of *Myrica esculenta*, is used to clean the teeth in case of toothache. Lemon juice, mixed with the powder of *Melia azedarach* bark, is used for the treatment of fever.		Cholera [26]
*Citrus × sinensis* (L.) Osbeck GA20171111NP041	Junar (T)	C	Fr	food med/gast,urogen	The ripe fruits are eaten, sometimes with chilly pepper. In case of hepatic and renal stones, 1–2 fruits are eaten a day, in small pieces during the day, for 15/25/30 days.		
**Santalaceae**							
*Osyris lanceolata* Hochst. & Steud. GA20171110NP035	Borsajini (T)	N	St St/Br	food med/musc	The stem portion near the ground is used to prepare a tea-like beverage. Used for dislocations and limb sprains. The stem portion near the ground is cut into small pieces, boiled for more than 3 h adding water; the filtered liquid is applied on the limbs banded with “Nepali paper”. The bandage is changed regularly for about one month. In case of bone fractures, the bark is cut into small pieces, boiled (even for more than 3 h), sometimes together with bark of *Senegalia catechu* and *Albizia julibrissin,* applied on the injured part and banded with “Nepali paper”.	[17,19]	Maternity problem [19]
*Viscum articulatum* Burm. f. GA20171109NP028	Khakhre bali (T)	W	Wp	med/musc	The crushed plant is applied on bone fractures and the part is banded with strips obtained from the *Urtica dioica* stem.	[24]	
**Sapotaceae**							
*Diploknema butyracea* (Roxb.) H.J.Lam GA20181015NP089	Chyuri (N)	W	Fr Br	med/derm,vet,fev	The crushed ripe fruits are applied externally for skin problems in humans and cattle. The bark of stem portion near the ground is dried, reduced to powder, mixed, and drunk with water or milk, to cure fever.		
**Saxifragaceae**							
*Bergenia ciliata* (Haw.) Sternb. GA20181017NP109	Pashanved (N), Bra mindhu (T)	W	Rh	med/gast,fev	Rhizome juice is drunk to treat stomach problems or the dried rhizome is chewed like candy throughout the day also to cure fever.	[16,19,24]	Piles, tumor, urinary problems, hearth, and respiratory diseases [16], maternity problem [17], back pain [25]
**Simaroubaceae**							
*Picrasma quassioides* (D. Don) Benn. GA20181018NP115	Nim kath (N)	W	Wood	med/fev,ant,musc,nerv	A typical recipe (*) is used to cure fever, internal fever, food poisoning, joint pain, migraine.		
**Solanaceae**							
*Solanum nigrum* L. GA20181012NP053	Camai (T)	W	Fr	med/ENT,card	Ripe fruits are consumed as much as possible, in case of tongue infections and piles.		Headache [23], wounds [24], malnutrition in children [26]
**Thymelaceae**							
*Daphne bholua* Buch.-Ham. ex D. Don GA20171110NP031	Lokta (N)	C	Br	handicr med/derm,ant,ENT,musc	The bark is used to make “Nepali paper”. “Nepali paper” is used to make bandages in case of skin problems, snake bites, bone fractures, swelling of the dental glands.	[16]	Fever, intestinal disorders, and parasites [16], sinusitis [19]
**Urticaceae**							
*Boehmeria virgata* (G.Forst.) Guill. subsp. *macrophylla* (Hornem.) Friis & Wilmot-Dear. (=*Boehmeria macrophylla* Hornem.) GA20171101NP016	Chalnesisnu (N)	W	L	vet	Leaves are nutritious cattle fodder.	[16]	Cuts and wounds [16,23]
*Boehmeria rugulosa* Wedd. GA20171101NP015	Bhlan chhing (T)	W	Br Wood	food handicr	Powdered bark is mixed with flour to make the bread softer and tastier. Wood is used for the production of religious masks and *teki*, container where butter (*ghee*) and yogurt are prepared.	[16]	Cuts and wounds, body pain [16]
*Urtica dioica* L. GA20171109NP027	Sisnu (N)	W	St	med/musc	Some strips obtained from the stem are used to wrap the limbs affected by bone fracture in association with the *Viscum articulatum.*	[17,26,27]	Galactogogue, diabetes, high pressure [17], fever, asthma, toothache, paralysis, uterine bleeding [19], rheumatism [25]
**Verbenaceae**							
*Lantana camara* L. GA20171101NP019	Polung (T)	N	Fr	food	Ripe black fruits are eaten by children as snack.		
**Xanthorrhoeaceae**							
*Aloe vera* (L.) Burm.f. GA20171109NP024	Ghyukumari (N)	C	L	med/derm,met,gast	The leaves are rubbed on burned skin. To treat jaundice, the leaf juice, or the filtered liquid of the leaves, crushed and boiled for 2–3 minutes is drunk. Some pieces of leaves are eaten in case of lack of appetite.	[19,20,26,27]	Cuts and wounds [23,24]
			Fl	dom	Flowers are used as an ornamental decoration.		
**Zingiberaceae**							
*Cautleya spicata* (Sm.) Baker GA20171110NP030	Pahelo Ausadhi (N) Jungli haldi (T)	W	Rh	med/gast,urogen	Rhizome juice is drunk once a day (morning) to treat constipation (*kabjiat*) and kidney stones. In case of gastric disorders, the juice is drunk once a week. Sometimes a second dose may be needed.		Conjunctivitis [26]
*Curcuma angustifolia* Roxb. GA20181013NP063	Jangali Haldi, Jangali Besar (N)	C	Rh	med/resp	Fumigations with powdered rhizome are useful to cure cold.		Cuts and wounds [24], stomach problems [26]
*Curcuma caesia* Roxb. GA20181017NP102	Mlang haldi (T)	C	Rh R	med/gast,mat	Used in case of loss of appetite. The cleaned rhizome is crushed and the juice is drunk with the addition of water, twice a day for 2 days. The root juice is drunk to treat postpartum bleeding.		Back pain [25]
*Curcuma longa* L. GA20181013NP064	Haldi, Besar (N)	C	Rh	med/met,fev	The raw rhizome is eaten to cure diabetes. The powdered rhizome is used for fumigations in case of fever.		Cough/cold, tonsillitis, swellings [16], gastritis [25]
*Zingiber officinale* Roscoe GA20181015NP083	Aduwa (N)	C	Rh	med/met	To cure goiter, the dried rhizome powder is mixed with the *Bauhinia variegata* bark powder and water, and applied to the throat twice a day (morning and evening).		Diarrhoea, sinusitis [23], cold and cough [27]

^a^ N—Nepali; Ne—Newari; T—Tamang; ^b^ W—wild; C—cultivated; N—naturalized; ^c^ Br—bark; L—leaves; Fl—flowers; Fr—fruit; Ys—young shoots; Lx—latex; R—root/rhizome; Sd—seeds; St—stem; Wp—whole plant; ^d^ agr—agriculture; dom—domestic use; handicr—handicraft; med—medicinal use; ant—antidote; card—cardiovascular; derm—dermatological; ENT—oral dental ENT; fev—fever; gast—gastrointestinal; genh—general health; mat—maternity; met—metabolic; musc—musculoskeletal; nerv—nervous system; resp—respiratory; urogen—urogenital; vet—veterinary; * Typical mix of plants: *Centella asiatica, Tagetes erecta, Mentha spicata, Artemisia indica*, *Oroxylum indicum*, *Oryza sativa*, *Picrasma quassioides*, *Oxalis corniculata, Drymaria cordata*.

**Table 3:** Taxonomic diversity of recorded plants species.

Family	Number of Species	Percent Value
Asteraceae	12	10.34
Fabaceae	7	6.03
Lamiaceae	6	5.17
Zingiberaceae	5	4.31
Amaranthaceae	4	3.45
Poaceae	4	3.45
Rutaceae	4	3.45
Anacardiaceae	3	2.59
Apocynaceae	3	2.59
Araceae	3	2.59
Euphorbiaceae	3	2.59
Lauraceae	3	2.59
Meliaceae	3	2.59
Urticaceae	3	2.59
Other 43 families	53	45.58
**Total: 57**	**116**	**100**

**Table 4:** Ailments included in each illness category.

Illness Category	Ailments	Number of Citations
Fever	Fever, high fever, malarial fever, typhoid fever, “internal” fever (feeling of higher level of heat inside the body)	56
Gastrointestinal	Stomach diseases, gastritis, vomiting, indigestion, loss of appetite, diarrhoea, dysentery, constpation (*kabjiat*), liver stones, abdominal pain, blood stool, flatulence	45
Musculoskeletal	Rheumatism, body pain, joint pain, joint trauma, joint swelling, ankle sprains, bone fracture, sprains of the limbs	30
Dermatological	Skin diseases, skin infections, cuts, wounds, pimples, boils, itchy pustules, burns	25
Antidote	Food and drink poisoning, snake bites	19
Respiratory diseases	Cough, cold, cooling diseases, sinusitis, rhinitis, throat irritations, breathing problems	17
Oral, dental, ENT (ear, nose, and throat)	Gums problem, mouth swelling, mouth infections, dry mouth, swelling of the dental glands, toothache, tongue infections, eye diseases, “red eyes” (conjunctivitis), nose swelling, ear infections, earaches	15
Metabolic	Blood purification, jaundice, diabetes, goiter	15
Nervous system	Migraine, headache, anxiety, insomnia	14
Maternal ailments	Difficulty in childbirth, infertility, menstrual disorders, postpartum bleeding	11
Urogenital	Urinary problems, bladder swelling, blood in the urine, kidney stones	9
Cardiovascular diseases	High blood pressure, blood circulation disorders, heart attack, piles	6
General health	Cancer, weakness, fatigue	2
Veterinary	Skin problems in cattle, reduced milk (agalactia) in buffaloes, general weakness	7
**Total**	**14**	**271**

**Table 5:** Plant parts used in the preparation of medicine.

Plant Parts	Use Citations	%
Root/Rhizome	64	23.61
Whole plant	62	22.88
Bark	27	9.96
Fruits	27	9.96
Seeds	19	7.01
Leaves	18	6.64
Flowers	17	6.27
Stem	14	5.16
Latex	10	3.69
Young shoots	8	2.95
Wood	5	1.84
**Total**	**271**	**100**

**Table 6:** Informants consensus factor (Fic) by categories of diseases, calculated only for several use reports ≥ 15.

Disease Category	Use Reports (Nur)	Number of Taxa (Nt)	Fic
Fever	56	29	0.49
Gastrointestinal	45	32	0.29
Dermatological	25	18	0.29
Metabolic	15	11	0.28
Oral dental ENT	15	11	0.28
Musculoskeletal problems	30	24	0.21
Antidote	20	17	0.16
Respiratory diseases	17	15	0.13

**Table 7:** Fidelity level (FL) value of medicinal plants against a given ailment category and main chemical compounds allegedly responsible for the therapeutic effects.

Medicinal Plant	Therapeutic Category	N_p_	N	FL Value (%)	Main Chemical Constituents
*Calotropis gigantea*	Dermatological	5	5	100	Lupeol present in the latex, with wound healing and antimicrobial properties [33].
*Drymaria cordata*	Fever	5	5	100	Saponins and related phytosterols, as well as phenols have been associated with antipyretic activity [34].
*Mangifera indica*	Gastrointestinal	3	3	100	Polyphenols and flavonoids from peel fruit with antinflammatory and antioxidant activities [35].
*Wrightia arborea*	Gastrointestinal	3	3	100	Not known.
*Oxalis corniculata*	Fever	4	5	80	Phenolic compounds with antibacterial activity [36].
*Centella asiatica*	Fever	3	4	75	Asiaticoside (triterpenoid) with antipyretic effects [37].
*Curcuma caesia*	Maternal ailments	3	4	75	Saponins with coagulating properties [38].
*Achyranthes bidentata*	Maternal ailments	4	9	44	Saponins with antinflammatory and antioxidant effects and promoting blood circulation [39].
*Achyranthes bidentata*	Fever	3	9	33	Saponins with antinflammatory and antioxidant effects and promoting blood circulation [39].

**Table 8:** Relative frequency of citation (RFCs) has been reported only for a value ≥ 0.1.

Plant Species	RFCs
*Achyranthes bidentata*	0.28
*Calotropis gigantea*	0.16
*Drymaria cordata*	0.16
*Artemisia indica*	0.13
*Centella asiatica*	0.13
*Curcuma caesia*	0.13
*Daphne papyracea*	0.13
*Oxalis corniculata*	0.13
